# Reading Self-Concept and Reading Anxiety in Second Grade Children: The Roles of Word Reading, Emergent Literacy Skills, Working Memory and Gender

**DOI:** 10.3389/fpsyg.2018.01180

**Published:** 2018-07-11

**Authors:** Tami Katzir, Young-Suk G. Kim, Shahar Dotan

**Affiliations:** ^1^Edmond J. Safra Brain Research Center for the Study of Learning Disabilities, Department of Learning Disabilities, University of Haifa, Haifa, Israel; ^2^Education, University of California, Irvine, Irvine, CA, United States

**Keywords:** reading, anxiety, self-concept, working memory, gender

## Abstract

**Background:** Most studies in the field of reading have focused on the linguistic and cognitive factors. Less is known about the affective aspects of reading in young readers, such as self-perceptions of reading, and reading anxiety.

**Aims:** This study aimed to shed light on the direct and indirect relations between reading and related skills (working memory, emergent literacy skills, word reading accuracy and rate, and gender) as sources of reading affect (reading self-concept and anxiety).

**Sample:** A total of 115 Hebrew speaking second graders participated in this study.

**Methods:** A set of measures assessing reading accuracy and rate, emergent literacy skills (phonological fluency, rapid automatized naming and working memory) and reading affect questionnaires (reading self-concept and reading anxiety) were administered to the participants.

**Results:** Path analysis was used as the primary analytic approach. Results indicated a negative moderate relation between reading self-concept and reading anxiety. The relations of working memory and emergent literacy to reading self-concept and reading anxiety were indirect via word reading accuracy and reading rate. Girls reported higher reading anxiety and lower reading self-concept, despite higher performance in reading accuracy and no difference in reading rate.

**Conclusion:** The current results support the importance of examining reading affect and potential sources of reading affect. Results suggest that reading self-concept and reading anxiety and their related skills should be considered in designing reading intervention and instructions.

## Introduction

Reading development involves complex interactive and dynamic processes among language, cognition, and affect. Most studies in the field of reading have focused on the linguistic and cognitive factors; and consequently less is known about the basis of affective aspects of reading in young readers, such as self-perceptions of reading (Kasperski et al., 2016). In the present study we address this gap in the literature by focusing on two affective aspects in reading, reading self-concept and reading related anxiety (reading anxiety hereafter), in young children. Specifically, we examined potential factors that influence reading affect by investigating the relations of working memory, emergent literacy skills, reading accuracy and rate, and gender to reading self-concept and reading anxiety.

### Reading Self-Concept, Reading Anxiety, and Reading

Reading self-concept is the overall self-perception of oneself as a reader (Conradi et al., 2014). It is related to reading motivation and to reading performance (Chapman and Tunmer, 1995, 2003; Conlon et al., 2006; Katzir et al., 2009; Kasperski et al., 2016), and the strength of these relationships increase with age (Chapman and Tunmer, 2003; Kasperski et al., 2016). For example, Conlon et al. (2006) showed that children’s perceptions of reading competence significantly accounted for variations in word identification, spelling and reading comprehension skills. In addition, children with more positive reading self-concept had higher performance on reading comprehension after accounting for their verbal ability and word reading (Katzir et al., 2009).

Another affect that has been related to reading skills is anxiety. Significant levels of general anxiety have been found to be negatively associated with reading skills for children (Merryman, 1974; Calvo and Carreiras, 1993; Tsovili, 2004; Grills-Taquechel et al., 2012, 2013, Grills et al., 2014; Plakopiti and Bellou, 2014). Studies with adult dyslexics have reported higher levels of general anxiety (Tsovili, 2004; Carroll and Iles, 2006; Plakopiti and Bellou, 2014), and academic and social anxiety (Boetsch et al., 1996; Carroll and Iles, 2006). For example, Meer et al. (2016) found that when asked to read aloud, adults with dyslexia exhibit higher level of physical arousal typically related to anxiety as measured by their Galvanic skin response when compared to typical readers. Taken together, the studies on children and adults with reading difficulties suggest that these readers show elevated level of anxiety. However, previous studies on reading and anxiety have typically focused on *general* anxiety in populations with severe reading difficulties (as seen earlier) or in the context of second language learning (L2) (Horwitz et al., 1986; Young, 1986, 1990, 1998; Horwitz, 2001; Brantmeier, 2005; Ghonsooly and Loghmani, 2012; Rajab et al., 2012; Tsai and Li, 2012; Mohammadpur and Ghafournia, 2015; Guimba and Alico, 2016). Less is known about specific worry and anxiety toward reading (reading anxiety^1^) in typically developing children (Piccolo et al., 2016).

### Sources Influencing Reading Self-Concept and Reading Anxiety

Even though the relationship between reading and reading self-concept has been consistently demonstrated, the mechanisms underlying this relationship remains to be clarified. Several alternative ideas have been proposed and examined. One hypothesis is that reading affect influences reading achievement (e.g., Baker and Wigfield, 1999; Bishop and Snowling, 2004; Conlon et al., 2006). For example, Attentional Control Theory (Eysenck and Derakshan, 2011) proposes that anxiety inhibits performance. It is well documented that anxiety reduces working memory capacity, and consequently people tend to retain less information from reading a text when they are anxious (Pratt et al., 1997; Derakshan and Eysenck, 1998; Eysenck et al., 2007). Similarly, Blair (2002) postulated that negative emotions of young children would lead them to focus on the object of their emotions rather than on the academic tasks they are asked to perform. Thus, negative emotions interfere with scholastic activities by reducing resources needed to integrate and attend to important details (see also Zhou et al., 2010; Owens et al., 2012) or by disrupting the effort students bring to their studies (Pekrun, 2006; Pekrun et al., 2009).

An alternative hypothesis is that children’s reading affect develops in response to reading experiences (Chapman and Tunmer, 2003). As many students judge their self-worth through academic success or failure, perceived failure on a test may increase fear of negative judgment and threat to self-worth (Covington, 1992; Denscombe, 2000; Chamberlain, 2012). Marsh (1986) suggested an internal/external frame of reference model (I/E model) for forming academic self-concepts. According to this model, students compare their levels of academic ability using two different but connected frames of reference: internal and external comparison processes. When it comes to reading, internal appraisal is related to one’s perceptions of difficulties or ease associated with reading tasks and experiences. External points of reference can be related to the teacher’s feedback, comparisons to peers on reading tasks, or experiences in the home (Katzir et al., 2009). Consistent with this view, Chapman et al. (2000) found that differences in self-concept between poor and average students were evident as early as a few weeks after commencing first grade and were also related to phonological awareness and letter naming knowledge. Building on these, Kasperski et al. (2016) recently hypothesized that reading self-concept develops as a function of reading skills. In particular, they hypothesized that reading rate, not accuracy, is particularly critical to forming students’ reading affect. According to their *rate-appraisal model*, individual differences in reading rate are highly salient factors for young developing readers, more so than reading accuracy, in the self-assessment of reading competence and may act as points of reference for the internal evaluation processes through which reading self-concept is shaped. Kasperski et al. (2016) explored the relationship of reading rate and accuracy to reading self-concept with Hebrew speaking children in grades two and three, and found that children’s performance on reading rate predicted reading self-concept after accounting for reading accuracy.

Similar to the idea about reading self-concept, the internal and external comparisons may also elevate one’s sense of worry about reading or reading anxiety, especially in struggling readers (Margalit and Zak, 1984) as reading anxiety is induced from past difficulties associated with reading (Zbornik, 1988, 2001; Zbornik and Wallbrown, 1991). Hinton et al. (2008) suggested that neuronal networks are built based on emotional responses to the task. Thus, if a child is asked to read aloud the stimulus ‘a’ (an initial neutral stimulus), and reading aloud is repeatedly paired with difficulties with performing the task and associated unpleasant situation (e.g., negative feedback), then a negative conditioned response to reading may occur. As a consequence, when the child is asked to read again, the act of reading will be associated with a sense of worry toward the reading process. In some cases, students fear making pronunciation errors or other mistakes while reading aloud in front of their peers (Ahmad et al., 2013). This speculation was supported by Grills-Taquechel et al. (2012) who investigated general anxiety and reading skills among first grade students and found that reading fluency (a composite of accuracy and speed) predicted separation anxiety symptoms and decoding skill was found to positively predict harm avoidance symptoms.

The third, final alternative hypothesis is a bidirectional relation between affect and reading skills (Jensen et al., 2013; Carey et al., 2016). According to this account, experiences with reading and reading related skills result in variation in reading self-concept and competence and anxiety, which, in turn, causes children’s responses to reading activities (affinity or anxiety). Children’s attitude toward reading activities, then, would result in variation in the amount of reading practice, which, in turn, relate to reading development.

Finally, gender might play a role in the development of reading self-concept and anxiety. From the early years of elementary school, girls are reported to have lower expectations and less confidence about future academic achievements than do boys (Frey and Ruble, 1987; Pressley et al., 1987). Moreover, girls were shown to be more affected by failure experiences than matched – ability boys (Licht and Dweck, 1984; Dweck, 1986). Later, in secondary education, girls further decline in academic self-concept at a faster rate than boys (De Fraine et al., 2007) despite the fact that girls tend to report enjoying reading more than do boys (Guthrie and Greaney, 1991), reading more books than do boys (Elley, 1994) and more motivated to read (Wigfield and Guthrie, 1997). When it comes to differential reading competence as a function of gender, high performance in word reading was associated with positive perceived competence in reading among girls whereas poorer achievers and boys had inflated self-perceptions such that they had positive perceived competence despite their low performance in word reading (Fives et al., 2014).

### Present Study

Previous studies have consistently indicated the relation between reading affect and reading performance. In the present study, our goal was to expand our understanding of reading and reading related skills (working memory, emergent literacy skills, word reading accuracy, word reading rate, and gender) as sources of reading affect (reading self-concept and anxiety). Under this overarching goal, we had several specific aims. First, we explored reading anxiety as well as reading self-concept as part of reading affect and examined their relations. Previous work on reading affect primarily focused on positive affect such as reading self-concept and competence (or efficacy) (as seen earlier) or the relations between general anxiety and reading with little attention to negative affect toward reading (reading anxiety). The second aim was to examine the relations of working memory and emergent literacy skills to reading self-concept and reading anxiety. Many studies have established the roles of working memory (e.g., Smith-Spark and Fisk, 2007; Berninger et al., 2008; Beneventi et al., 2010; Moll et al., 2014; Kim et al., 2017), phonological awareness (e.g., Wagner and Torgesen, 1987; Byrne, 1998; National Institute of Child Health, and Human Development [NICHD], 2000), and rapid naming (Swanson et al., 2003; Kirby et al., 2010; Manolitsis et al., 2011; Moll et al., 2014) in word reading skills across languages. If reading affect emerges from reading and reading related experiences, then, foundational skills of reading (i.e., working memory and emergent literacy skills) might relate to reading self-concept and reading anxiety. An important corollary to this research question is paths of relations. If working memory and emergent literacy skills are related to reading self-concept and reading anxiety, then, are their relations direct or primarily indirect via word reading skills? To our knowledge, only one study examined the relation of emergent literacy skills to reading affect – Kasperski et al. (2016) found that one of the emergent literacy skills, rapid automatized naming, was indirectly related to reading self-concept via reading rate.

The third aim was to clarify the relations of word reading accuracy versus reading rate to reading affect (reading self-concept and reading anxiety). As noted earlier, Kasperski et al. (2016) hypothesized a salient role of reading rate for reading self-concept and found supporting evidence. We replicate and extend Kasperski et al.’s study by examining the relations of word reading accuracy and rate to reading self-concept *and* reading anxiety. If reading experiences influence how the child feels toward reading, both accuracy and rate may play roles in positive emotions and negative emotions related to reading (reading self-concept and anxiety). The current study will focus on the Hebrew orthography, which in the first stages of reading acquisition is characterized as a shallow orthography. In this orthography children achieve high proficiency rate in accuracy around the end of second grade (Geva et al., 1997; Share and Levin, 1999; Shany et al., 2012).

The last aim of the present study was to extend previous studies on the relation of gender reading to reading affect. Specifically, we investigated how gender is related to reading self-concept and reading anxiety after accounting for word reading accuracy and rate. A previous study reported that boys tended to have an inflated reading competence (Fives et al., 2014). In the present study, we examined whether boys and girls have differential reading self-concept and reading anxiety after accounting for word reading skills (accuracy and rate), working memory, and emergent literacy skills.

We hypothesized that working memory, rapid naming, and phonological awareness would be indirectly related to reading self-concept and reading anxiety via word reading accuracy and word reading rate, because children’s perceptions and appraisal on their reading ability would be based on explicitly reading tasks. In other words, children are likely to perceive reading words in print as a reading task compared to manipulating sounds (i.e., phonological awareness), and therefore, the point of reference for appraisal of their reading skills would be their performance on ‘reading’ tasks, not emergent literacy skills or working memory. We also hypothesized that word reading rate would be particularly important to reading self-concept based on a previous study (Kasperski et al., 2016). However, we did not have an *a priori* hypothesis about whether reading accuracy versus rate would be differentially related to reading anxiety versus reading self-concept, given little previous work. We also anticipated that female students would have lower reading self-concept and higher anxiety even after accounting for word reading accuracy, word reading rate, rapid naming, phonological awareness, and working memory.

## Materials and Methods

### Participants

Participants in this study were 115 second grade learners, 65 boys (56.52%) and 50 girls (43.48%). Children were from two schools in northern Israel. Based on the Israeli Ministry of Education’s school SES index which classifies schools based on parents’ education, family income, school location and immigration and neighborhood, the first school was from a medium-high socioeconomic background (decile 2) and the other was from low socioeconomic background (decile 9). No differences were found on all baseline measures between the two schools. The participants’ age ranged 7–9 years (*M* = 7.71; *SD* = 0.44). Data were collected at the end of the academic year. All participants had normal or corrected to normal vision and hearing. Due to the fact that diagnosis of learning disabilities in Israel begins in third grade and above, no child had a diagnosis of learning disability.

### Measures

#### Working Memory

The Digit Span test in Wechsler Intelligence Scale for Children-Revised (WISC-R) was used. Participants were asked to repeat strings of digits in the same order, and in reversed order. There were 16 strings in each task, and the test was stopped when the participant failed to recall two strings of the same length. Strings recalled correctly on each test scored one point. Standard score was calculated. Reliability is reported to be 0.87 (Williams et al., 2003).

#### Phonological Awareness

The phonemic fluency test (Kavé, 2005) was used where students was asked to say as many Hebrew words as possible beginning with the letter *bet*, except for names of people and places. The number of words retrieved in 1 min was their score. Reliability (α) was reported to be 0.90 (Kavé, 2005).

#### Rapid Automatized Naming

The alphabet subtest of the rapid automatized naming (RAN-L) was used from “Alef Ad Taf” (Shany et al., 2006). This subtest consisted of five Hebrew letters, 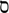 (s), 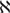 (a), 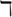 (d), 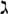 (g), and 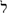 (l), and each repeated randomly 10 times. The child was asked to name an array of 50 letters arranged in five rows of 10 letters each as fast as he could and no specific instructions were given in regard to accuracy. Self-corrections and errors were noted by the experimenter for the purposes of qualitative observation, but these were very rare. Following the works of Denckla and Rudel (1976) and Wolf and Denckla (2005), the total time taken to name the letters was the student’s score. Alternate form reliability was reported to be 0.74 in second grade (Shany et al., 2006).

#### Reading Self-Concept

In the present study we used the competence subscale from the Reading Self-concept Scale (Chapman and Tunmer, 1995). The competence subscale contains 10 items (e.g., can you work out what a story means?). The experimenter read each item aloud and the participant was required to respond to each question on a scale ranging from 1 (no, never) to 5 (yes, always) and higher scores reflected a more positive self-concept. Cronbach’s α was 0.84.

#### Reading Anxiety

A questionnaire was adapted on the basis of the Abbreviated Math Anxiety Scale (AMAS) (Hopko et al., 2003). The questionnaire was adjusted to measure reading anxiety among second grade students. Similar to the test developed by Zbornik (1988), the examiner first explained to the children that the reading anxiety is a test to find out how children feel about reading, but there are no right or wrong answers. Subsequently, the examiner read the statements to the children and they answered how much they agree with the statement, circling strongly agree, agree, not sure, disagree, or strongly disagree in the booklet. For example, the statement, “How worried are you when you think about 1 day before an upcoming math test?” was modified to “How concerned are you when you think about an upcoming literacy lesson?” The questionnaire included nine statements (e.g., “think of literacy lessons,” “begin to study a new topic in literacy classes”). Replies were given on a five-point Likert-type scale, ranging from 1 (never concerned) to 5 (always concerned) such that higher scores reflected higher anxiety. Cronbach’s α was 0.83.

### Procedures

The study was approved by the Chief Scientist of the Ministry of Education in Israel as well as the Research Ethics Committee of the Department of Education at the University of Haifa. Before testing, written informed consent was obtained from the parents of the participants. The tests were individually administered over two sessions by trained graduate students in a quiet room at the school. Each session lasted approximately 20–30 min.

### Data Analytic Strategy

**FIGURE 1:**
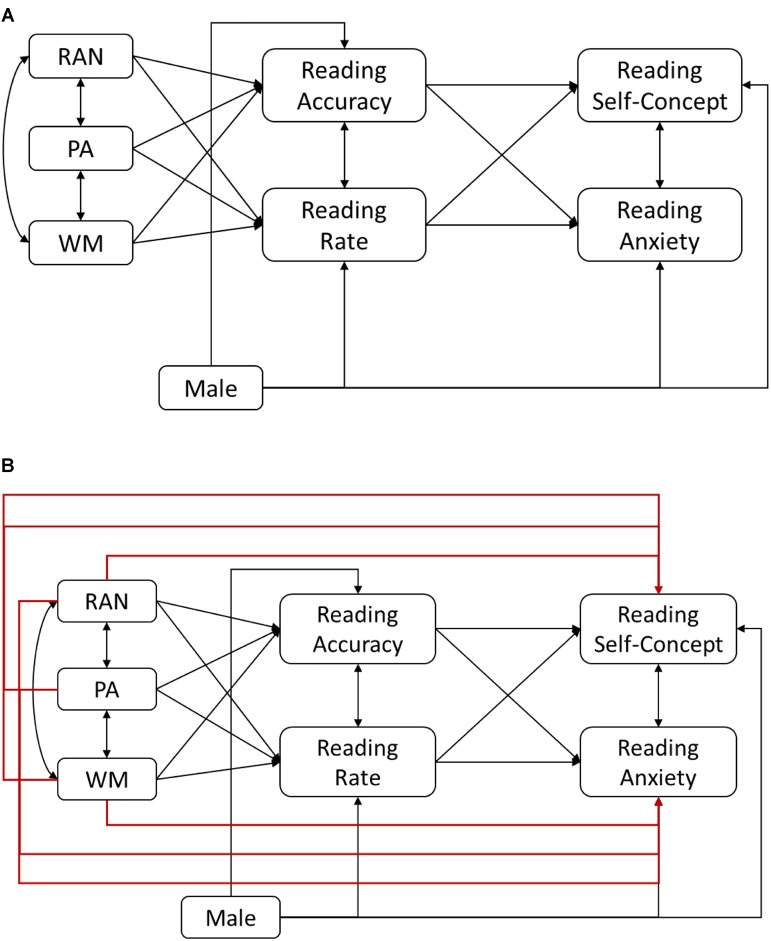
Two alternative models of the relations of working memory, phonological awareness (PA), rapid automatized naming (RAN), reading accuracy, reading rate, and gender to reading self-concept and reading anxiety. In **(A)**, reading accuracy and reading rate completely mediate the relations of RAN, PA, and working memory to reading self-concept and reading anxiety whereas in **(B)**, they mediate the relations partially.

Path analysis was used as the primary analytic approach to address the research questions. The relation between reading self-concept and reading anxiety was examined in the bivariate correlation and in the path analysis. To examine the research question about the relations of working memory, emergent literacy skills, to reading self-concept and reading anxiety, two the two alternative models are shown in **Figures 1A,B** were compared. In **Figure 1A** model, word reading accuracy and word reading rate were hypothesized to completely mediate the relations of rapid automatized naming, phonological awareness, and working memory to reading self-concept and reading anxiety. In **Figure 1B** model, these variables were hypothesized to be directly related to reading self-concept and reading anxiety over and above word reading accuracy and rate (i.e., partial mediation model). In these models, reading self-concept and reading anxiety were allowed to covary because the primary question is not the nature of relation between reading self-concept and reading anxiety, but instead the nature of relations of predictors to these outcomes. Because these two models were nested, model fit differences were tested with chi-square difference. Note that these models also address the research question about the relations of reading accuracy and reading rate to reading self-concept and anxiety because rapid automatized naming, phonological awareness, and working memory were hypothesized to predict word reading accuracy and word reading rate, which, in turn, were hypothesized to predict reading self-concept and reading anxiety. The relations of gender to word reading accuracy, word reading rate, reading self-concept, and reading anxiety were also examined in these models (see **Figure 1**). Note that in preliminary analysis gender was not related to working memory and emergent literacy skills and thus, the paths from gender to working memory and emergent literacy skills were not included in these path models. To address the research question about the predictive relations between reading self-concept and reading anxiety, again two alternative path models were fitted to the data. In the first model (**Figure 3**), reading self-concept was hypothesized to predict reading anxiety. In the second model (**Figure 4**), reading anxiety was hypothesized to predict reading self-concept.

Overall model fits were determined by the following multiple indices: chi-square statistic, comparative fit index (CFI), Tucker-Lewis index (TLI), root mean square error of approximation (RMSEA), and standardized root mean square residuals (SRMR). Excellent model fits are characterized by RMSEA values below 0.08, CFI and TLI values = or >0.95, and SRMR = or <0.05 (Hu and Bentler, 1999). TLI and CFI values >0.90 and SRMR = or <0.10 are considered acceptable (Kline, 2005).

## Results

### Descriptive Statistics

Descriptive statistics are presented in **Table 1**. Students’ performances on the tasks had sufficient variation around the means. In the reading self-concept, the mean was 32.16 with standard deviation (SD) of 7.04. In the digit span task, which had normative information, the mean standard score was in the average range (*M* = 11.03, *SD* = 2.57). Students, on average, read with high accuracy (*M* = 84%; *SD* = 12%). Students read, on average, 35 words/min. Distributional properties in terms of skewness and kurtosis were all within the acceptable range (±3).

**Table 1:** Descriptive statistics.

Variable	Mean (SD)	Min–Max	Skewness	Kurtosis
RAN letters	40.63 (8.25)	26–69	0.85	0.87
Phonological awareness	6.20 (2.49)	0–13	-0.04	0.07
Digit span (raw)	9.55 (2.04)	5–15	0.16	-0.30
Digit span (SS)	11.03 (2.67)	5–18	0.19	-0.20
Reading accuracy (percent accurate)	84.34 (11.70)	23–100	-2.20	7.61
Reading rate	34.96 (19.49)	11.50–142.50	2.18	8.27
Reading self-concept	32.16 (7.04)	9–40	-1.06	0.59
Reading anxiety	14.43 (6.59)	9–35	1.33	1.01


### Correlations

Bivariate correlations are presented in **Table 2**. Reading self-concept and reading anxiety were negatively and moderately related (*r* = -0.58, *p* < 0.001). Reading anxiety was negatively related to all the other variables (-0.16 ≤*r*s ≤-0.28). Directions of other variables were in expected directions. Rapid automatized naming was weakly and negatively related to the other variables (-0.08 ≤*r*s ≤ -0.36). Phonological awareness and working memory (digit span) were also weakly to somewhat moderately related to other variables (0.15 ≤*r*s ≤ 0.42).

**Table 2:** Correlations between variables.

Variable	1	2	3	4	5	6
1. RAN letters	–					
2. Phonological awareness	-0.22^∗^	–				
3. Digit span	-0.19^∗^	0.28^∗∗^	–			
4. Reading accuracy	-0.08	0.22^∗^	0.42^∗∗∗^	–		
5. Reading rate	-0.36^∗^	0.34^∗∗∗^	0.36^∗∗∗^	0.28^∗∗^	–	
6. Reading self-concept	-0.18	0.23^∗^	0.15	0.31^∗∗^	0.39^∗∗∗^	–
7. Reading anxiety	-0.19^∗^	-0.26^∗∗^	-0.19^∗^	-0.16	-0.28^∗∗^	-0.58^∗∗∗^


*^∗^*p* < 0.05; ^∗∗^*p* < 0.01; ^∗∗∗^*p* < 0.001.*

### The Relations of Working Memory, Emergent Literacy Skills, Word Reading Accuracy and Rate, and Gender to Reading Self-Concept and Reading Anxiety

In order to address the question about direct and indirect relations of working memory and emergent literacy skills to reading self-concept and reading anxiety, two alternative models are shown in **Figures 1A,B** were fitted. Model fit for the complete mediation model (**Figure 1A**) was good: χ^2^(9) = 12.39, *p* = 0.19, CFI = 0.97, TLI = 0.94, RMSEA = 0.057, SRMR = 0.046. The partial mediation model (**Figure 1B**) also had good fit to the data: χ^2^(3) = 3.56, *p* = 0.31, CFI = 1.00, TLI = 0.97, RMSEA = 0.04, and SRMR = 0.03. Chi-square difference test showed no difference between these two models (Δχ^2^ = 8.83; Δdf = 6, *p* = 0.18), and therefore, a more parsimonious model, the complete mediation model (**Figure 1A**) was selected as the final model.

**FIGURE 2:**
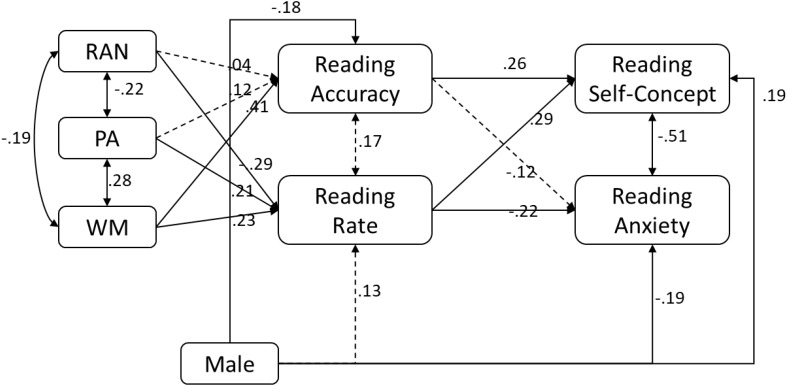
Results of path model where reading self-concept and reading anxiety are covaried.

Standardized beta weights of the complete mediation model (**Figure 1A**) are presented in **Figure 2**. Rapid automatized naming, phonological awareness, and working memory were all independently related to word reading rate (-0.29 to 0.23, ps < 0.02). Furthermore, working memory was independently related to word reading accuracy. Word reading accuracy and word reading rate were not related to each other (0.17, *p* = 0.06) once phonological awareness, rapid automatized naming, and working memory were accounted for. Gender was negatively related to word reading accuracy such that male students had lower word reading accuracy (-0.18, *p* = 0.03), but there was no difference in word reading rate between male and female students (0.13, *p* = 0.10). Despite this lower word reading accuracy, male students had statistically higher reading self-concept (0.19, *p* = 0.02), and lower reading anxiety (-0.19, *p* = 0.04).

Reading self-concept and reading anxiety were moderately and negatively related (-0.51, *p* < 0.001) after accounting for the variables in the model. Furthermore, word reading accuracy (0.26, *p* = 0.003) and reading rate (0.29, *p* < 0.001) were both independently related to reading self-concept whereas for reading anxiety, only reading rate (-0.22, *p* = 0.02), not reading accuracy, was related.

### Predictive Relations Between Reading Self-Concept and Reading Anxiety

**FIGURE 3:**
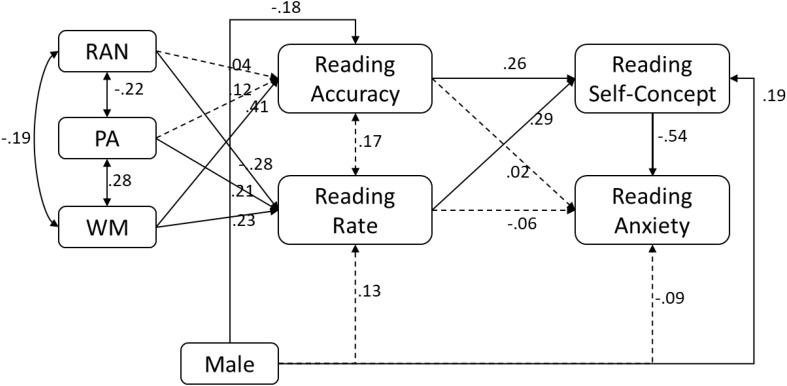
Results of path model where reading self-concept predicts reading anxiety.

**FIGURE 4:**
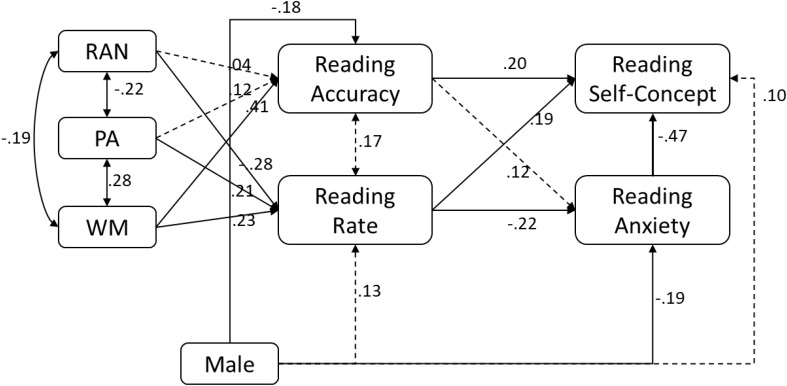
Results of path model where reading anxiety predicts reading self-concept.

In order to examine the predictive nature between reading self-concept and reading anxiety as well as their mediating roles, two models were fitted to the data. In the first model (**Figure 3**), reading self-concept was hypothesized to predict reading anxiety where in the second model (**Figure 4**), reading anxiety was hypothesized to predict reading self-concept. Results for the first model is shown in **Figure 3**. Not surprisingly, reading self-concept predicted reading anxiety with moderate magnitude (-0.54, *p* < 0.001). What is notable is that gender was positively related to reading self-concept (0.19, *p* = 0.02), but was not related to reading anxiety (-0.09, *p* = 0.29), indicating that reading self-concept completely mediates the relation of gender to reading anxiety. Furthermore, reading self-concept completely mediated the relation of reading rate to reading anxiety as reading rate was not related to reading anxiety (-0.06, *p* = 0.50) after controlling for its relation to reading self-concept.

When reading anxiety was hypothesized to predict reading self-concept (**Figure 4**), reading anxiety predicted reading self-concept (-0.47, *p* < 0.001). The gender effect on reading self-concept was no longer statistically significant (0.10, *p* = 0.17), once its relation to reading anxiety was accounted for (-0.19, *p* = 0.04). Furthermore, reading rate was related to both reading anxiety (-0.22, *p* = 0.02) and reading self-concept (0.19, *p* = 0.01) such that reading anxiety partially mediated the relation of reading rate to reading self-concept.

## Discussion

In spite of their clear relevance, students’ emotions in school settings have been largely neglected in education research (Pekrun et al., 2002). This is especially true in the domain of reading. Research on reading typically focuses on cognitive and linguistic factors, with less attention to the role of affective factors. Individuals’ emotions were shown to be linked to academic success (Pekrun et al., 2017), and most of the available studies concentrated on positive feelings toward learning. Enjoyment of learning, for instance, was shown to be linked to achievement (Götz and Hall, 2013). Similarly, motivation and reading self-concept were related to school achievement in different ages (Katzir et al., 2009; Schaffner and Schiefele, 2013; Pekrun et al., 2017). However, the role of negative emotions such as reading anxiety has rarely been studied in typical young readers. Moreover, the links among emergent literacy skills, gender, reading self-concept and reading anxiety have not been studied.

The present study adds several important, unique findings to the extant literature. First, we found that reading anxiety was moderately and negatively correlated with reading self-concept – a measure that has also been linked to reading motivation. This pattern is consistent with findings on math anxiety and math self-concept (e.g., Pajares and Miller, 1994). Children who exhibit a lower sense of competence in reading are those who are afraid of reading and may subsequently develop avoidance. These findings in young children, as early as the end of second grade, underscore the importance of evaluating a wide spectrum of emotions, both positive and negative, in developing readers. As the initial stages of reading acquisition may be particularly more challenging for some children, the long term effects of these feelings should be examined and monitored over time. Addressing these feelings early on may help diminish their impact over time.

Second, we found that the relations of working memory and emergent literacy to reading self-concept and reading anxiety are primarily indirect via word reading accuracy and reading rate. A large body of literature has shown the relation of working memory, rapid automatized naming, and phonological awareness to reading skills (e.g., Wagner and Torgesen, 1987; Byrne, 1998; National Institute of Child Health, and Human Development [NICHD], 2000; Berninger et al., 2008; Beneventi et al., 2010; Kirby et al., 2010; Manolitsis et al., 2011; Moll et al., 2014; Kim et al., 2017). Then, students’ emergent literacy skills may be related to reading affect. However, when we explicitly tested alternative models, an indirect relations model was supported, indicating that although working memory and emergent literacy skills are important foundations for word reading development, and they are not directly related to reading self-concept and reading anxiety. These findings also suggest that children’s appraisal or perceptions of their own reading ability is likely to be based on reading tasks, not emergent literacy skills.

Third, the findings revealed differential relations of word reading accuracy and reading rate to reading self-concept versus reading anxiety. For reading self-concept, both reading accuracy and reading rate were independently related, suggesting that children’s experiences with accuracy and rate are likely to influence their reading self-concept. This finding diverges from Kasperski et al. (2016) study which found a unique relation of rate, but not accuracy, to reading self-concept. The discrepancy might be due to several differences between the studies, including grade of the children, and included variables in the statistical model. The children in the present study were in second grade whereas those in Kasperski et al.’s study were in second and third grade. Therefore, children in Kasperski et al.’s study may have been in a more advanced stage of reading development at which point reading rate may be a more salient factor for self-appraisal of reading skills. When the outcome was reading anxiety which was not investigated in previous studies, reading accuracy was not related. The relation of reading rate to reading anxiety, on the other hand, varied to some extent as a function of the directionality of hypothesized relations between reading self-concept and reading anxiety. When the directionality is hypothesized from reading self-concept to reading anxiety (**Figure 3**), the effect of reading rate on reading anxiety was indirect, completely mediated by reading self-concept. In other words, reading rate influences how one feels about as a reader, which then, influences worry and anxiety about reading. When the directionality was from reading anxiety to reading self-concept, reading rate uniquely predicted reading anxiety, indicating that reading anxiety partially mediates the relation of reading rate to reading self-concept. That is, reading rate does contribute to the child’s negative feelings (worry and anxiety) associated with reading, but it also makes a direct contribution to reading self-concept over and above reading anxiety.

Overall, these findings suggest that reading rate may serve as the basis of self-appraisal of one’s reading skills that influences reading self-concept and reading anxiety. This is in line with a previous finding that reading rate was the strongest and single predictor of reading self-concept for those in second and third grade (Kasperski et al., 2016) as well as studies in foreign language learning – students with high foreign language anxiety have slower speed of processing in reading tasks, than do non-anxious readers (MacIntyre and Gardner, 1994; Loghmani and Ghonsooly, 2012). Thus, in line with Marsh’s model of I/E feedback as the basis of self-evaluation (Marsh, 1986), in the case of reading, the self is centrally impacted by the sense of speed. Therefore, for young children, an internal and external source of positive and negative self-evaluation may be reading speed. However, unlike Kasperski et al. (2016) study, we found the role of reading accuracy in reading self-concept, which indicates that children’s sensitivity to their reading accuracy (whether they are prone to reading errors) is likely to be part of their self-appraisal of their reading ability.

The present findings are from children learning to read in Hebrew. Hebrew, in its pointed version (with diacritic marks which children use until fourth grade), is a shallow orthography, including one-to-one correspondence between letters and sounds (Geva et al., 1997). Hence, learning to read pointed Hebrew is less demanding than opaque languages (e.g., English) and is characterized by 90% average accuracy in the end of second grade (Geva et al., 1997; Share and Levin, 1999). This pattern may lead to different roles of reading accuracy and rate on reading affect in reading Hebrew compared to other languages.

There are several directions for future studies. First, a future study should capture processes of children’s self-appraisal and investigate whether the roles of reading accuracy and reading rate vary as a function of children’s developmental phase of reading – both reading accuracy and reading rate might influence one’s appraisal during the beginning phase when children are more likely to make decoding errors whereas during the more advanced phase, errors are limited and more palpable aspect of reading performance to children might be reading speed. Second, due to the differences in the developmental trajectories of reading accuracy and rate across languages, future studies should examine the roles of both reading accuracy and rate in reading affect across languages.

Third, the present findings are also revealing about the relation between gender and reading affect. Gender based differences have been widely studied in many academic domains across different age groups (e.g., Hill et al., 2016). In the academic context, it is socially assumed that boys show higher abilities in math and science, while fewer girls have reading difficulties (Quinn and Wagner, 2015). In fact, in this study, girls were more accurate than boys in their word reading. In light of this, it is reasonable to hypothesize that girls would have lower reading anxiety and higher reading self-concept. However, girls reported significantly higher reading anxiety and lower reading self-concept (see **Figures 3**, **4**), indicating that girls are more critical of or overly sensitive to their reading compared to boys. This finding diverges from previous studies which based on questionnaires reported no differences in reading self-concept among third grade boys and girls (Marinak and Gambrell, 2010). On the other hand, these results are consistent with those of Frenzel et al. (2007) who concluded that although fifth grade girls did not differ from boys in math achievements, the girls reported significantly higher anxiety, hopelessness, and shame, and less enjoyment and pride than boys. These gender differences in the academic anxiety measure for typical children are in line with earlier works on math anxiety (Wigfield and Meece, 1988; Frenzel et al., 2007), test anxiety (Pintrich and de Groot, 1990), and second language anxiety(Alkhateeb, 2014).

A plausible explanation for these findings may be related to the fact that girls generally have lower confidence with regard to academic achievements (Wigfield and Meece, 1988; Frenzel et al., 2007). Most importantly, a future fine-grained look at gendered responses to reading experiences (success and failure) is needed. It may be that girls are more critical of themselves in response to their own internal feedback as well as external feedback (Marsh, 1986). Another important possibility that requires a future investigation is the potential social factors in the differential development of reading self-concept and reading anxiety as a function of gender. The higher reading anxiety among girls may be the result of gender stereotypes, which are known to influence children as early as second grade (Jameson, 2014). Stereotypes of females as helpless, passive, and dependent result in girls’ reduced feelings of control and promote internal worry in girls (Pomerantz et al., 2002).

Although not the primary focus in this study, a couple of other findings are worth noting. First, reading rate and accuracy were not related after accounting for the included emergent literacy skills in this sample of second grade Hebrew speaking children. This finding is in line with previous studies which showed a dissociation between rate and accuracy in reading development in Hebrew for second, fourth and sixth graders (Shany et al., 2012). Second, rapid naming and phonological awareness were not independently related to word reading accuracy whereas rapid naming was related to reading rate. These findings are consistent with other findings on first grade Hebrew speaking children (Shechter, 2017)^2^.

### Limitations and Future Directions

The following several limitations are worth noting. First, our focus was on the relations of reading and emergent literacy skills to reading affect. However, as noted earlier, theoretical accounts and empirical evidence suggest a potential bidirectional relation between affect and academic achievement (Jensen et al., 2013; Carey et al., 2016). Therefore, future longitudinal studies as well as experimental studies should explore the bidirectional nature of the relationship. Second, it is possible that reading anxiety is part of a more general structure such as trait anxiety or achievement anxiety. Future studies should examine the connection between reading anxiety and other anxieties. In addition, as stated in the introduction, children’s self-concept is affected and defined by social comparison. A better understanding of the potentially challenging feelings associated with specific aspects of reading can provide educators with knowledge and understanding about feelings experienced in the context of academic tasks. Reading instruction, combined with teacher awareness of students’ emotional and behavioral difficulties, can lead to reading success (Atkinson et al., 2002). Third, we focused on word reading accuracy and reading rate in relation to reading self-concept and reading anxiety for children in grade 2. However, given that reading is developmental phenomenon with the goal of successful reading comprehension, it would be important to understand the presence of reading anxiety in all reading stages. A longitudinal study examining reading and reading related skills to reading affect from the beginning to advanced phases of reading would be informative. In addition, although none of the children in the present study were diagnosed with reading difficulties because of the diagnosis timeline in Israel (grade 3 and above), it is apparent that there are children in the sample whose reading accuracy and reading rate are very low (see **Table 1**). An examination of potentially differential patterns of relations for students with typically developing reading skills versus those with reading difficulties was beyond the scope of the present study. However, children with reading difficulties or dyslexia might show more pronounced relations of reading with reading affect due to greater difficulties and associated higher anxiety (e.g., Carroll and Iles, 2006; Plakopiti and Bellou, 2014; Meer et al., 2016), and therefore, future studies are warranted.

Another important future direction is intervention work. Previous studies have shown that reading motivation is malleable such that a 4-week motivational engagement program has recently been linked to significantly higher reading comprehension of seventh grade students (Guthrie and Klauda, 2014). While a substantial amount of research has focused on reading motivation in children (Bates et al., 2016), future work should work on other aspects such as reading self-concept and anxiety in terms of intervention.

## Conclusion

Although most of prior research has focused on the cognitive and linguistic aspects of reading development, growing body of literature indicates the importance of reading affect and its interactions with cognitive and linguistic factors. Overall, our findings support the relations of reading skills and gender to reading affect such as reading self-concept and reading anxiety. These add to the extant literature and indicates the importance of emotional aspects along with the linguistic and cognitive ones.

## Author Contributions

TK contributed in conceptual framework, task development, and writing the article. Y-SK contributed in data analysis, conceptual framework analysis, and writing the article. SD contributed in data collection and writing the article.

## Conflict of Interest Statement

The authors declare that the research was conducted in the absence of any commercial or financial relationships that could be construed as a potential conflict of interest.

## Footnotes

1 The term, anxiety, is typically used in a clinical context as a diagnosed phobia that may occur in abnormal situations or in pathological samples. In this paper we refer to reading anxiety as worry or fear about reading which is a continuous construct found in typical readers as well as struggling readers.

2 In this study, first graders’ phonological skills were less related to reading pointed Hebrew, i.e., the transparent version of the Hebrew orthography (see Shany et al., 2012 for a discussion of transparent and deep versions of the Hebrew orthography).
